# *Acinetobacter baumannii* Virulence Is Mediated by the Concerted Action of Three Phospholipases D

**DOI:** 10.1371/journal.pone.0138360

**Published:** 2015-09-17

**Authors:** Julia Stahl, Holger Bergmann, Stephan Göttig, Ingo Ebersberger, Beate Averhoff

**Affiliations:** 1 Department of Molecular Microbiology and Bioenergetics, Institute of Molecular Biosciences, Goethe University Frankfurt, Frankfurt am Main, Germany; 2 Department of Applied Bioinformatics, Institute for Cell Biology and Neuroscience, Goethe University Frankfurt, Frankfurt am Main, Germany; 3 Institute of Medical Microbiology and Infection Control, University Hospital Frankfurt, Goethe University, Frankfurt am Main, Germany; Centre National de la Recherche Scientifique, Aix-Marseille Université, FRANCE

## Abstract

*Acinetobacter baumannii* causes a broad range of opportunistic infections in humans. Its success as an emerging pathogen is due to a combination of increasing antibiotic resistance, environmental persistence and adaptation to the human host. To date very little is known about the molecular basis of the latter. Here we demonstrate that *A*. *baumannii* can use phosphatidylcholine, an integral part of human cell membranes, as sole carbon and energy source. We report on the identification of three phospholipases belonging to the PLD superfamily. PLD1 and PLD2 appear restricted to the bacteria and display the general features of bacterial phospholipases D. They possess two PLDc_2 PFAM domains each encompassing the HxKx_4_Dx_6_GS/GGxN (HKD) motif necessary for forming the catalytic core. The third candidate, PLD3, is found in bacteria as well as in eukaryotes and harbours only one PLDc_2 PFAM domain and one conserved HKD motif, which however do not overlap. Employing a markerless mutagenesis system for *A*. *baumannii* ATCC 19606^T^, we generated a full set of PLD knock-out mutants. *Galleria mellonella* infection studies as well as invasion experiments using A549 human lung epithelial cells revealed that the three PLDs act in a concerted manner as virulence factors and are playing an important role in host cell invasion.

## Introduction

*Acinetobacter baumannii* is one of the most successful and most threatening nosocomial (hospital-acquired) Gram-negative bacteria implicated in septicaemia, meningitis, wound infections, urinary tract infections and pneumonia [1,2]. In recent years, the National Nosocomial Infection Surveillance System (NNIS) revealed a substantial increase in *A*. *baumannii*-associated nosocomial pneumonia cases. Overall, they make up 5–10% of the intensive care unit (ICU)-acquired pneumonia cases in the United States [3–6]. Moreover, *A*. *baumannii* ranks 10^th^ among the organisms causing monomicrobial bloodstream infections [1,4]. Besides its steady increase in resistance against a broad spectrum of antibiotics the pronounced resistance to desiccation of *A*. *baumannii* is also highly worrying [1,7,8]. The persistence and virulence of *A*. *baumannii* can be attributed to a broad spectrum of factors. These include biofilm formation, quorum sensing, resistance to desiccation, possession of powerful iron acquisition systems and the production of virulence factors such as phospholipases C and D [8–14]. In addition, *A*. *baumannii* displays a number of traits conveying adaptation to the human as a host. For example, it can adhere to, colonize and invade human epithelial and endothelial cells and displays human serum resistance [8–10,14–17]. However, despite the increasing threat of *A*. *baumannii* surprisingly little is known about the molecular mechanisms of host adaptation.

The utilization of carbon sources available in the host is one of the prerequisites for the persistence of pathogens in the human host. The human body is a rich source of nutrients and it has been shown that different pathogens use different carbon sources during host colonization, such as lactic acid, mucus sugars, amino acids and cholesterol, just to mention of few [18]. Phospholipids are also very abundant in the human host since they are the major building blocks of biological membranes making them also good candidates as carbon and energy source. Phosphatidylcholine (PC) is particularly abundant in eukaryotic membranes accounting for 50% of all phospholipids [19]. In the lung the prevalence even increases up to 80% [20] and PC is also the major phospholipid in tracheobronchial secretions where it has significant impact in wettability of the mucus [21]. There is strong evidence that phosphatidylcholine serves as nutrient source during lung-infections by pathogens like *Pseudomonas aeruginosa* [22]. Taking into account that ventilator-associated pneumonia is the most prevalent manifestation of *A*. *baumannii* infection [23], high abundance of PC in the mucus raised the question whether PC serves as nutrient source for *A*. *baumannii* in the human host.

Phospholipases (PL) are key enzymes, essential for the metabolism of PC and have been studied in a diverse spectrum of bacteria [24–26]. Three classes of phospholipases (PLA, PLC, PLD) have been defined by the site of cleavage. PLA hydrolyzes fatty acids from the glycerol backbone. Upon PLC cleavage, the phosphorylated head group is released from the phospholipid and PLD cleaves off only the head group. Both, release of the phosphorylated headgroup and release of the polar head group can affect the stability of host cell membranes. In addition, phospholipases can also interfere with cellular signalling by generating second messengers like phosphatidic acid, which are capable of modulating host immune responses [24–26]. Information about the distribution of phospholipases in different *A*. *baumannii* strains and their role in virulence is scarce. So far, only two phospholipases, one PLC and one PLD have been identified as virulence factors in *A*. *baumannii* ATCC 17978 and in the clinical isolate 98-37-09, respectively [11,12]. Mutation studies revealed an involvement in cytotoxicity and epithelial cell invasion, respectively, and suggested a potential role of the two enzymes in virulence.

In this study, we identified three *pld* genes in *A*. *baumannii* ATCC 19606^T^ and performed a thorough bioinformatic and experimental characterization of the corresponding proteins. We show that *pld1* and *pld2* evolved through a recent gene duplication, most probably in the last common ancestor of *Acinetobacter*. In contrast, *pld3* appears substantially older and we provide initial evidence that it may share evolutionary descent with the cardiolipin hydrolase ZUC in *Drosophila*. Furthermore, PLD3 shows an exchange of a highly conserved aspartate by a tyrosine in the HKD active site motif of the PLDc_2 domain, otherwise a hallmark of the PLD superfamily. For functional characterization of the PLDs by mutation studies, we developed a markerless mutagenesis system to generate a whole set of *pld* knock-out mutants. Bacterial competition studies, infection of *Galleria mellonella* and invasion of A549 human lung epithelial cells revealed that the three PLDs do not play a role in bacterial competition but act in a concerted manner in infection and invasion of eukaryotic host cells.

## Materials and Methods

### Organisms and Cultivation

*A*. *baumannii* ATCC 19606^T^ was grown at 37°C in Luria Bertani medium (LB) or mineral medium (MM) with 20 mM acetate as carbon source as described earlier [27]. Kanamycin was added when appropriate to a final concentration of 50 μg/ml. *E*. *coli* DH5α was grown at 37°C in LB medium. When appropriate, kanamycin or ampicillin were added to a final concentration of 20 μg/ml and 100 μg/ml, respectively. *Pseudomonas putida* 548.C8 [28] was grown in LB medium with 20 μg/ml kanamycin at 30°C.

### Growth Experiments

Growth experiments with *A*. *baumannii* ATCC 19606^T^ wildtype and mutants were performed using 500 ml Erlenmeyer flasks filled with 100 ml medium (LB or MM + 20 mM acetate) and incubated shaking (130 rpm) at 37°C. Growth experiments were started by inoculation of a stationary phase culture into fresh medium to OD_600_ of 0.15. Growth was followed at 37°C by analyzing the optical density at 600 nm.

For growth on different carbon sources, *A*. *baumannii* was inoculated in a test tube containing 5 ml MM with either 20 mM acetate, glycerol or choline or MM with either 0.5% PC or 0.5% palmitate as sole carbon source. Growth experiments were started by 1:10 dilution of an appropriate overnight pre-culture, grown on the corresponding carbon source. Growth on acetate, glycerol and choline was monitored by increase of the OD_600_ over time. Growth on PC or palmitate was determined by plating serial dilutions on LB agar and CFU counting at different time points. In each case, MM without any carbon source was used as negative control, whereas growth on acetate was used as positive control.

### Data Collections and Data Sources

To determine the phyletic profile of PLDs across the entire tree of life we first compiled the TOL set comprising protein collections from 1281 bacteria, 123 archaea and 204 eukaryotes that are available from the Orthology Matrix project web sites (OMA, Release 16, Mach 2014, http://cbrg-oma-test.ethz.ch/oma/archives/). These data were complemented by the set of proteins encoded in the genome of the *A*. *baumannii* type strain ATCC 19606^T^ as provided by NCBI GenBank (http://www.ncbi.nlm.nih.gov/genome/browse/representative/). Our second data set (ACI set) was designed to provide a high-resolution presence-absence pattern of PLDs in the genus *Acinetobacter*. The ACI set comprises the proteins from the 14 *Acinetobacter* species represented in the TOL set together with the proteins from 54 additional *Acinetobacter* strains provided by NCBI GenBank (http://www.ncbi.nlm.nih.gov/genome/browse/representative/). The full taxon lists of both the TOL (S1 Table) set and the ACI (S2 Table) set are available as supporting information. Please note that putative products of annotated pseudogenes are not explicitly considered in the analyses.

### Annotation of Functional Domain Content

We annotated Pfam-A domains [29] in the PLD proteins employing *hmmscan* from the HMMER3 package [30] (http://hmmer.janelia.org). Further functional features and signatures in the protein sequence were annotated with interproscan (http://www.ebi.ac.uk/Tools/pfa/iprscan5/) using default settings. Domain architectures were visualized and arranged with Domosaics v. 0.95 [31]. The presence of signal sequences was analyzed using SinglaP 4.1 [32].

### Ortholog Search

For the detection of orthologs in both the TOL and the ACI set we used the targeted ortholog search tool HaMStR v. 13.2 [33] (http://sourceforge.net/projects/hamstr). We seeded the ortholog searches for the three proteins, PLD1, PLD2, and PLD3, respectively, with the corresponding protein sequences from *A*. *baumannii* ATCC 19606^T^. We then used an initial HaMStR search to first compile a manually curated core set of orthologs for each of the three proteins. In the curation step we verified that the domain architecture of the identified orthologs resembles that of the respective seed proteins. The final core sets comprised proteins from the following bacterial species: *Paenibacillus terrae* strain HPL-003, *Yersinia pestis* strain CO92, *Chlamydia trachomatis* strain B, *Acinetobacter calcoaceticus* strain PHEA-2, *Helicobacter pylori* strain PeCan4 and *A*. *baumannii* strain ATCC 19606^T^. Subsequently, we aligned the sequences in the three core sets with MAFFT-LINSI [34] and used the alignments to train a profile hidden Markov model (pHMM) with hmmbuild from the HMMER3 package [30]. The resulting refined pHMM served then as input for the second ortholog search in the full compilation of bacterial, archaeal and eukaryotic gene sets.

### Phylogenetic Analysis

We aligned the amino acid sequences in the individual orthologous groups using MAFFT-LINSI [34]. For the resulting alignments, ProtTest 3.2 [35] identified the VT model [36] modeling rate across sites with a gamma distribution (+G) as the best-fitting substitution model. Maximum likelihood tree reconstruction was performed with RAxML 8.1.9 [37] using the PROTGAMMAVT model for amino acid sequence evolution. For statistical support, 100 nonparametric bootstrap replicates were computed. A combined tree for all three ortholog groups was reconstructed using FastTree 2.1.7 [38], and branch support was assessed with a local Shimodaira-Hasegawa test [39] as implemented in FastTree.

### Markerless Mutagenesis

A *sacB/kanR*-cassette was amplified from the genome of *A*. *baylyi* ADP1 *hptA*::*sacB/kanR* [40] using the primer combination sacB/kanR_for and sacB/kanR_rev (S3 Table). A 1825 bp fragment spanning the *pld2*-gene (1632 bp), as well as 45 bp of the up- and 145 bp of the downstream region, respectively, was amplified from the genome of *A*. *baumannii* ATCC 19606^T^ using pld2_up_for and pld2_do_rev primers. The fragment was cloned into *Kpn*I and *Pst*I sites of pBIISK (Invitrogen). A central 316 bp fragment of *pld2* was deleted by restriction with *Eco*RI and *Hinc*II followed by Klenow fill-in and subsequent ligation, resulting in plasmid pBIISK_pld2_updown. The *sacB/kanR* cassette was inserted into the *Sma*I-site of this plasmid by blunt end ligation, resulting in pBIISK_sacB/kanR_pld2_updown. To generate the constructs for *pld1* and *pld3*-mutation the *sacB/kanR* cassette was inserted into the *Eco*RV site of pBIISK (Invitrogen) by blunt end ligation leading to pBIISK_sacB/kanR. For each construct ~1500 bp of the up- and downstream region of *pld1* and *pld3* were amplified from the genome of *A*. *baumannii* ATCC 19606^T^ using the following primer combinations: pld1_up_for and pld1_up_rev (*pld1* upstream-region), pld1_do_for and pld1_do_rev (*pld1* downstream region), pld3_up_for and pld3_up_rev (*pld3* upstream region), pld3_do_for and pld3_do_rev (*pld3* downstream region). Fragments containing up- and downstream regions of *pld1* and *pld3* were cloned into pBIISK_sacB/kanR using *Pst*I, *Bam*HI and *Not*I resulting in the vectors pBIISK_sacB/kanR_pld1_updown and pBIISK_sacB/kanR_pld3_updown.

To generate electro-competent *A*. *baumannii* cells, an overnight culture of *A*. *baumannii* ATCC 19606^T^ was inoculated into 400 ml fresh LB medium and grown until OD_600_ of 0.45–0.5. The cultures were cooled down on ice for 10 minutes and washed 4 times with sterile, ice-cold mili-Q H_2_O, followed by one washing step in sterile, ice-cold 10% glycerol. Cell pellets were resuspended in 500 μl 10% glycerol. 50 μl of the *A*. *baumannii* cell suspensions were used as recipient for electroporation of plasmid DNA (2–3 μg DNA). Electroporation was performed at 2.5 kV, 200 Ώ and 25 μF in 2 mm pre-cooled electroporation cuvettes. After electroporation, 1 ml pre-warmed SOC medium (2% tryptone; 0.5% yeast extract; 10 mM NaCl; 2.5 mM KCl; 10 mM MgCl_2_; 10 mM MgSO_4_; 20 mM Glucose) was added. Cells were incubated for 1 hour at 37°C, and subsequent transformants were selected on LB-Agar + 50 μg/ml kanamycin. Integration of the plasmid was monitored by colony PCR using the primer combinations ctr_for + do_rev and up_for + ctr_rev (S3 Table).

Correct integrants were subjected to counter-selection overnight at 37°C in 5 ml LB medium with 10% sucrose. Appropriate dilutions of the cultures were plated onto LB agar medium with 10% sucrose and incubated at 37°C for 12 h. Single colonies were tested for kanamycin sensitivity by replica plating onto LB agar ± 50 μg/ml kanamycin. Kanamycin sensitive colonies were subjected to PCR analyzes using the primers ctr_for + ctr_rev (S3 Table). Double and triple mutants were obtained by insertion and segregation of the plasmids comprising the truncated phospholipase genes of interest into single or double *pld* mutants.

### Interbacterial Competition Assay

The interbacterial competition between wild-type (WT) or *pld*-mutants and *E*. *coli* DH5α or *P*. *putida*, respectively, was analyzed as described earlier for competition between *A*. *nosocomialis* and *E*. *coli* [41,42]. *A*. *baumannii* and *E*. *coli* DH5α pET28a were grown at 37°C in 20 ml LB medium to an OD_600_ of 1.5–2.0. *P*. *putida* 548.C8 [28] was grown at 30°C in 20 ml LB medium to an OD_600_ of 1.5–2.0. Subsequently, OD_600_ was adjusted to 0.4 (~ 2 * 10^7^ CFU/ml) and *A*. *baumannii* and *E*. *coli* DH5α cells or *A*. *baumannii* and *P*. *putida* cells were mixed in a 10:1 ratio. Twenty μl of this mixture were spotted onto LB agar and incubated at 37°C for 4 hours. Spots were excised from the agar plate and resuspended in 500 μl 0.9% NaCl. Serial dilutions were plated onto LB agar + 50 μg/ml kanamycin to select for surviving *E*. *coli* or *P*. *putida* cells.

### *Galleria mellonella* Infection Assay

*Galleria mellonella* caterpillars (larvae of the greater wax moth) were obtained by a local provider. Caterpillars were preselected by melanization, size and movement in response to touch. *A*. *baumannii* wild-type and *pld* mutant cells were grown on LB medium until late exponential growth phase, washed with 0.9% NaCl and adjusted to an OD_600_ of 2. In each set of infection, 16 caterpillars were used to test each bacterial strain in one test. Caterpillars were whipped in 70% EtOH for external disinfection and 10 μl (~ 1 * 10^6^ CFU/ml) of the bacterial suspension were injected into the last left proleg. As control served a set of untreated caterpillars as well as a set of caterpillars in which 10 μl of 0.9% NaCl were injected. Caterpillars were incubated at 37°C in the dark for 4 days. Caterpillars were considered as dead if they did not respond to gentle probing. All experiments were repeated at least 4 times and experiments in which 2 or more caterpillars in one of the control groups died were not considered.

### Adhesion and Invasion Assays

Human lung epithelial A549 (ATCC CCL-185) cells were grown to confluence in six well plates in RPMI 1640 medium (Merck Millipore, Darmstadt, Germany) supplemented with 10% fetal calf serum (FCS) at 37°C and 5% CO_2_. *A*. *baumannii* was grown in LB medium overnight at 37°C. Stationary cultures were washed once in RPMI medium prior to addition of 5x10^7^ bacteria to each well, corresponding to a multiplicity of infection (MOI) of approximately 200 bacteria per A549 cell. The plates were incubated for 5 hours at 37°C in 5% CO_2_ to allow adhesion and invasion of bacteria, as described earlier [43]. For analysis of invasion, A549 cells were washed three times with Dulbecco’s phosphate-buffered saline (PBS). Afterwards, 2 ml RPMI containing 500 μg/ml gentamicin were added to each well for 2 hours to eliminate all extracellular bacteria. After three washing steps with PBS, A549 cells were lyzed by addition of 800 μl distilled H_2_O for 15 min, to release invaded bacteria from the disrupted A549 cells. Then, 200 μl 5x PBS was added and serial dilutions were plated on LB-Agar for determination of CFU/ml. For analysis of adhesion, A549 cells were infected as described above, washed three times with PBS and lyzed with H_2_O to release all internal and adherent bacteria. All invasion and adhesion assays were performed in duplicates and were repeated at least three times.

### Statistical Analysis

Paired t-tests were performed with GraphPad Prism 6 (GraphPad Software Inc., San Diego, CA, USA). Values are presented as mean ± SEM. Growth rate was determined with GraphPad Prism 6 using exponential growth equation.

## Results

### Use of Phosphatidylcholine as sole Carbon and Energy Source

**Fig 1:**
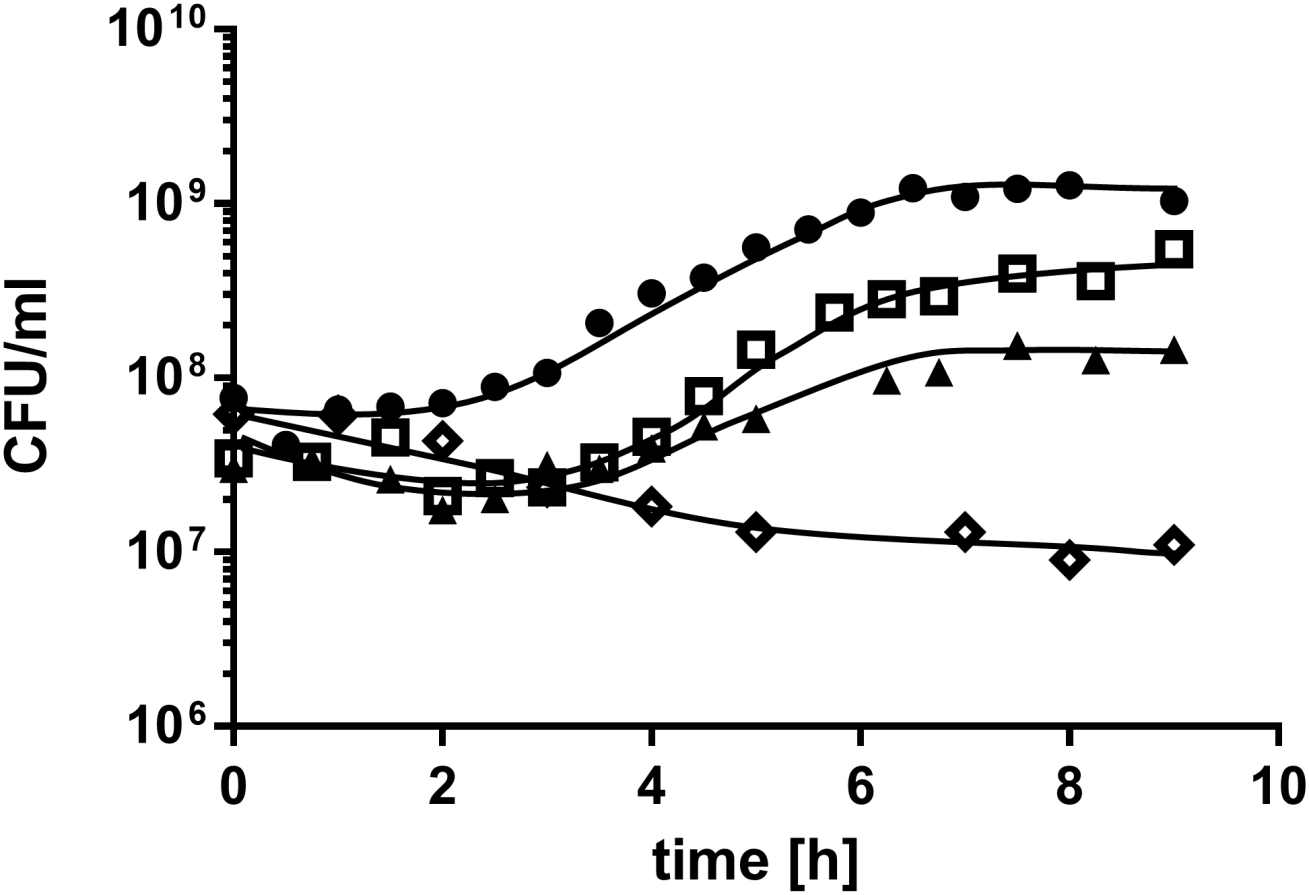
Growth of *A*. *baumannii* ATCC 19606^T^ on PC, palmitate and acetate. *A*. *baumannii* ATCC 19606^T^ was grown in 5 ml MM with either 0.5% PC (triangles), 0.5% palmitate (squares) or 20 mM acetate (circles) as sole carbon source. A culture without carbon source (diamonds) was used as negative control. Growth was followed by plating appropriate dilutions on LB-agar plates at the indicated time points. The amount of colony forming units (CFU) was determined after incubation at 37°C overnight.

Growth experiments on mineral medium (MM) containing phosphatidylcholine revealed that PC serves as carbon and energy source for *A*. *baumannii* ATCC 19606^T^ with a growth rate of 0.378 ± 0.028 h^-1^ and a final cell density of ~ 1.4∙10^8^ CFU/ml (Fig 1). To elucidate which of the PC cleavage products were used as carbon source by *A*. *baumannii* we tested growth in MM with glycerol, fatty acids and choline as sole carbon and energy sources. Neither glycerol nor choline alone could serve as sole carbon and energy source since no increase in optical density was observed after incubation for 2 days. In contrast, palmitate, which is one of the most common fatty acids in PC, was used as carbon and energy source by *A*. *baumannii* with a growth rate of 0.545 ± 0.057 h^-1^ and a final cell density of ~ 5∙10^8^ CFU/ml (Fig 1). As positive control for all growth experiments served MM with 20 mM acetate, resulting in a growth rate of 0.586 ± 0.001 h^-1^ and a final cell density of ~ 1.1∙10^9^ CFU/ml. As negative control served MM without carbon source. No increase in optical density as well as CFU was observed after the first transfer in MM without carbon source.

### Screen for Phospholipases D in the Genome of *A*. *baumannii*

In previous studies, we could show that *Acinetobacter baylyi* ADP1 oxidizes choline after growth on MM containing choline and identified a potential choline oxidation pathway in the genome of A. *baylyi* ADP1 [44]. A homologous pathway, including a choline dehydrogenase (*BetA*), a betaine-aldehyde dehydrogenase (*BetB*), a potential regulator (*BetI*) as well as two choline transporters (*BetT1* and *BetT*2) was also detected in the genome of *A*. *baumannii* ATCC 19606^T^. Therefore, we concluded, that *A*. *baumannii* can also oxidize choline [44], raising the question whether *A*. *baumannii* exhibits PLDs, which catalyze the liberation of choline from PC. We screened the genome of *A*. *baumannii* ATCC 19606^T^ for the presence of potential *pld* genes and identified three candidates: The open reading frame HMPREF0010_00607 (*pld1*) spans 1527 bp and encodes a hypothetical PLD1 protein with a deduced molecular mass of 58.8 kDa. HMPREF0010_03706 (*pld2*) spans 1632 bp and encodes a hypothetical phospholipase PLD (PLD2) with a molecular mass of 62.4 kDa. PLD2 is identical to the recently published PLD of *A*. *baumannii* 98-37-09 [12]. The third candidate, HMPREF0010_02731 (*pld3*), has a predicted size of 1464 bp and encodes a hypothetical 55 kDa protein.

**Fig 2:**
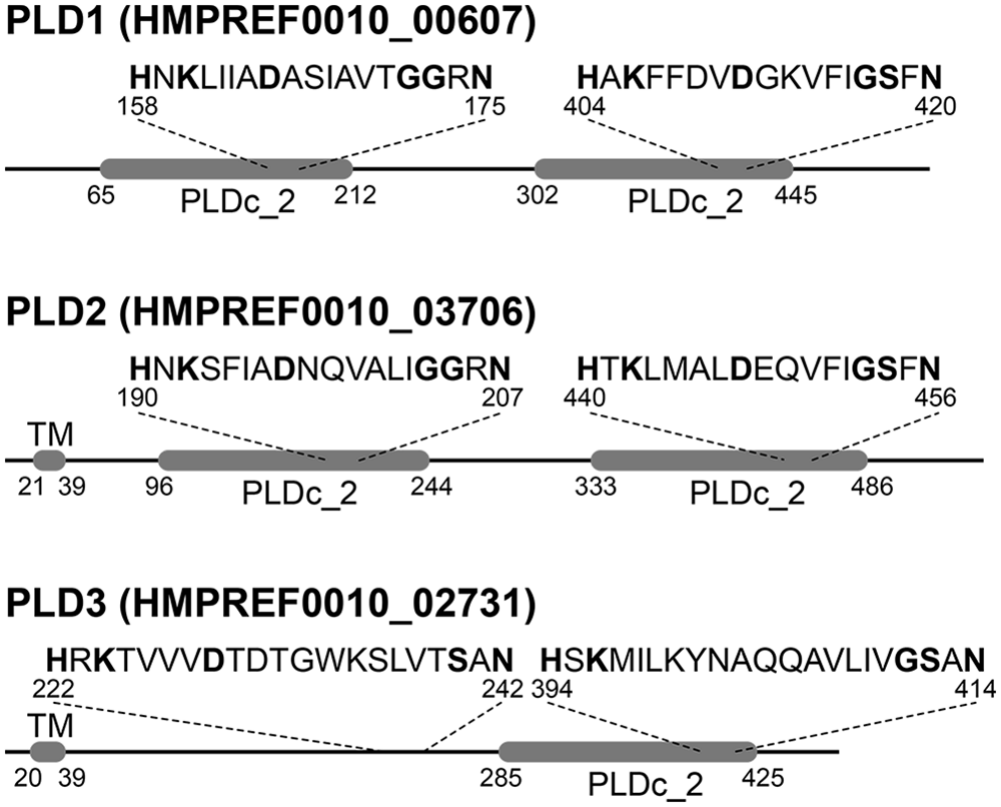
Domain architecture of the three *A*. *baumannii* ATCC 19606^T^ phospholipase D candidates. PLD1 and PLD2 each possess PLDc_2 Pfam domains (PF13091). Each domain harbors the conserved motif characteristic for the active center of type D phospholipases. PLD3 has only a single PLD domain encompassing a HKD-like motif (HxKx_4_Dx_6_GS/GGxN) where the functionally relevant aspartate is replaced by a tyrosine. A second canonical HKD motif is present in the N-terminal half of the protein and outside the context of a PLDc_2 domain. While both, PLD2 and PLD3, appear membrane bound proteins, PLD1 lacks a transmembrane domain (TM).

Subsequently, we characterized the putative PLDs *in silico*. A screen for conserved domains and signatures in the three proteins revealed that PLD1 and PLD2 each harbor two phospholipase D-like domains PLDc_2 (PF13091). Embedded in both domains we detected the 28 amino acid long phospholipase D phosphodiesterase active site motif (PF00614; Fig 2). This motif spans the HxKx_4_Dx_6_GS/GGxN pattern that has been described as the catalytic center of the phospholipases [45]. Interestingly, the third candidate, PLD3, deviates from this domain architecture. It features only one phospholipase D-like domain and one active site motif, which however do not overlap. While the active site is located in the central part, the PLDc_2 domain is located C-terminal of PLD3. Notably, a subsequent screen of the PLD3 sequence with reduced stringency revealed a second HKD-like motif overlapping with the C-terminal PLDc_2 domain. However the typically highly conserved aspartic acid (D) is replaced in this motif by a tyrosine (Y) (Fig 2). Our further annotation revealed that none of the *pld* genes encodes a signal sequence indicative for protein export. Furthermore PLD1 lacks a transmembrane region, whereas PLD2 and PLD3 both possess a hydrophobic domain which is predicted to represent a membrane anchor.

### Phyletic Distribution and Evolutionary History of *A*. *baumannii* Phospholipases D

First, we analyzed the distribution of PLD candidates in the genus *Acinetobacter* by a targeted ortholog search in the 68 bacterial strains of the ACI set. PLD1 orthologs are present in 60 taxa, while PLD2 and PLD3 orthologs are present in all of the 68 analyzed taxa (S2 Table). This observation indicates that PLD1 can be absent in individual strains of the genus *Acinetobacter* due to gene loss, a loss of function, or pseudogenization—the latter can be observed for *A*. *baumannii* strain AYE and its PLD1 ortholog. Please note, that the two other PLD1 pseudogenes described by Vallenet *et al*. 2008 [46] are not represented in the AYE protein sets downloaded from the OMA web pages. The resulting presence/absences matrix is given in S2 Table.

**Fig 3:**
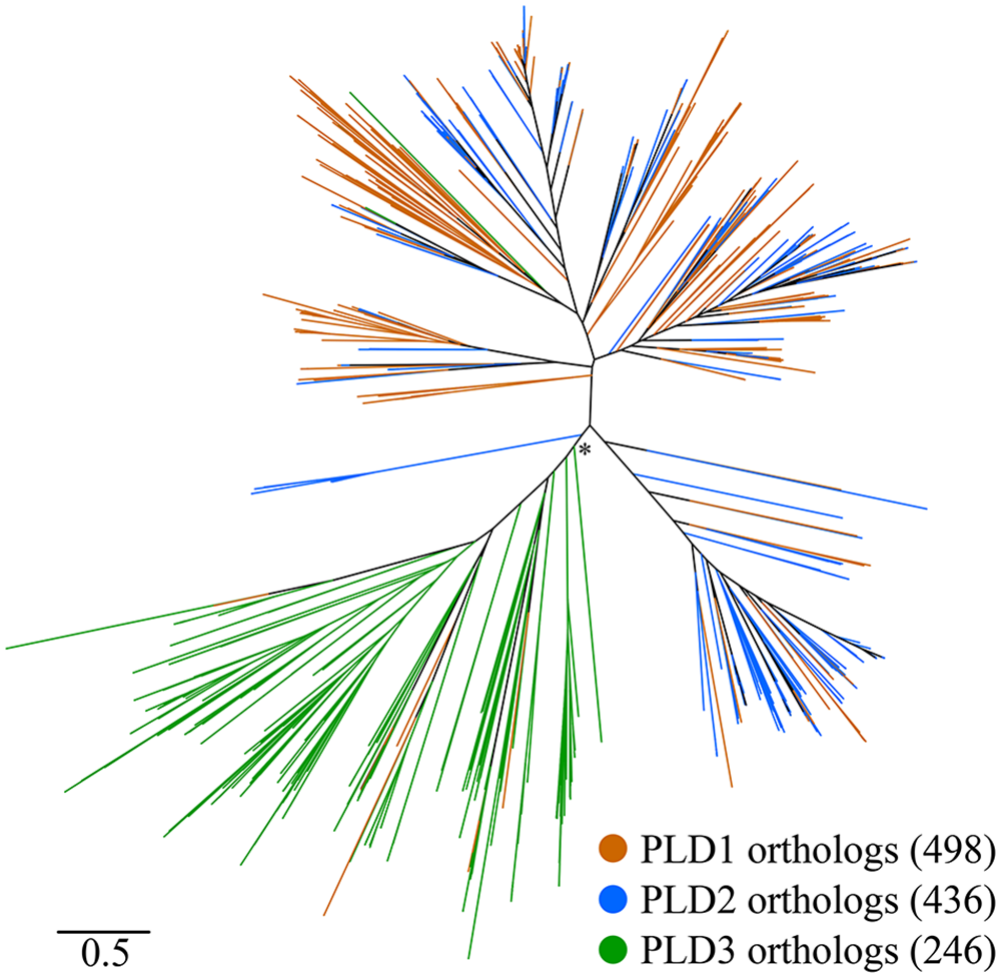
Phylogenetic tree combining the orthologous groups of PLD1, -2, and -3. The tree demonstrates the intertwined evolutionary relationships of orthologs assigned to *A*. *baumannii* PLD1 and PLD2, respectively. In contrast, PLD3 sequences form a well separated clade suggesting an evolutionary origin distinct from that of PLD1 and PLD2. The * denotes a local branch support of 0.83.

**Fig 4:**
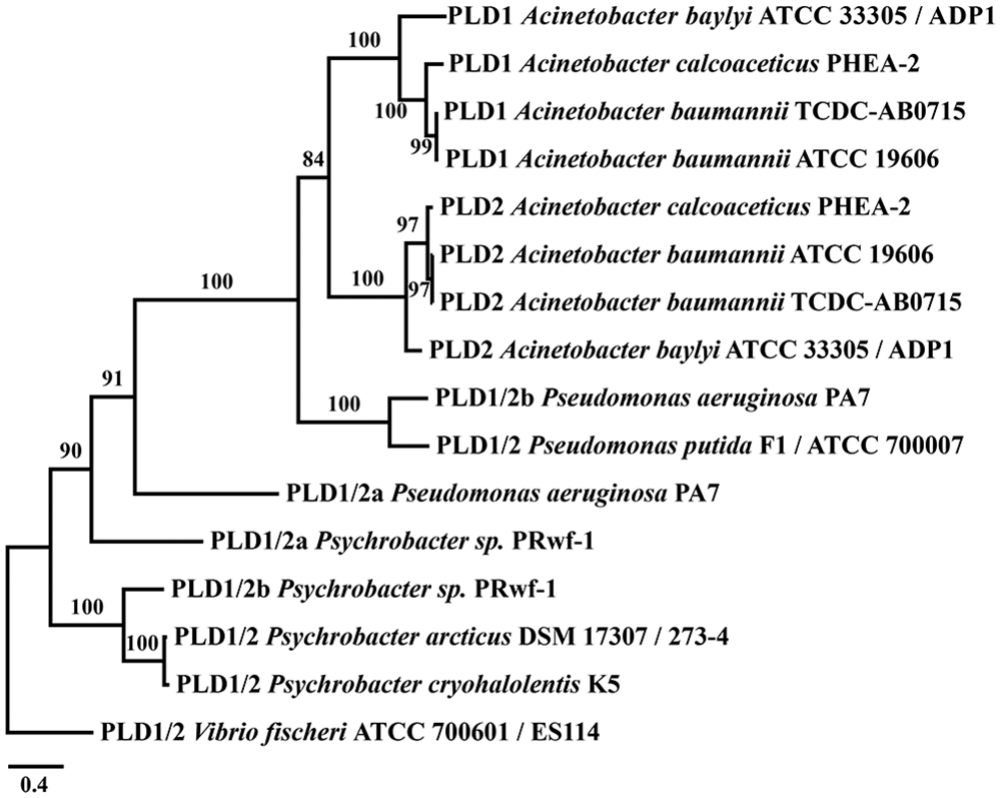
Evolutionary relationships of *Acinetobacter baumannii* PLD1 and PLD2. The tree reveals that PLD1 and PLD2 emerged through a recent gene duplication that occurred after the split of *P*. *aeruginosa* and possibly in the last common ancestor of the genus *Acinetobacter*. Orthologs whose split pre-date the diversification of PLD1 and 2 were renamed to PLD1/2. Branch labels represent percent bootstrap support.

Second, we characterized the phylogenetic profile and the evolutionary relationships of the three PLD candidates. To this end, we first performed a targeted ortholog search in the TOL set comprising proteins from 1,281 bacteria, 124 archaea, and 204 eukaryotes. The results are summarized in S4 Table and the detected orthologs are provided in the S1 File. Both PLD1 and PLD2 are widely distributed across bacteria with orthologs present in 498 and 436 taxa, respectively. In archaea and eukaryotes orthologs could be identified in only 10 (PLD1) and 3 (PLD2) taxa respectively. Given the sparse and scattered phyletic distribution of these candidates the orthology assignments are most likely spurious. This suggests that both proteins are confined to bacteria. The phylogenetic profile of the third protein, PLD3, differs substantially from that of the other two PLDs. Though orthologs were also found throughout the analyzed bacterial clades, PLD3 is with only 187 detected orthologs less common in bacteria than both, PLD1 and PLD2. However, and again in contrast to the other two proteins, its prevalence in the eukaryotes, and here particularly in the animals is marked (S4 Table). Analyses of the phylogenetic tree revealed that orthologs assigned to the *A*. *baumannii* PLD1 and PLD2 genes, respectively, are well mixed in the tree (Fig 3). Such an intermingled topology indicates a rather recent evolutionary separation of the corresponding *A*. *baumannii* genes. A subsequent maximum likelihood tree reconstruction with a taxon sampling tailored to shed further light on this issue confirmed this hypothesis and narrowed the gene duplication event to have happened within the *Moraxellaceae* lineage (Fig 4 and S5 Table). In contrast to the entwined relationships of PLD1 and PLD2 orthologs, our PLD tree also indicates that, with very few exceptions, all PLD3 orthologs from the three domains of life reside in a single clade to the exclusion of the other PLDs (Fig 3). Determination of the Pfam domain architecture revealed that the vast majority (178) of PLD3 orthologs share the presence of only a single PLDc_2 domain as a common feature (S1 Fig and S3 File). Moreover, archaean sequences are distributed across the reconstructed phylogeny suggesting several independent evolutionary origins of these sequences. In contrast, the eukaryotic sequences form, with two exceptions, a monophyletic clade and the corresponding subtree resembles by and large the eukaryotic species phylogeny. This is consistent with a single evolutionary origin of the eukaryotic PLD3 sequences. As one possibility they may share a common descent with their bacterial orthologs from an evolutionarily ancient gene in the last universal common ancestor. Alternatively, they might have been horizontally introduced into the eukaryotic lineage. In this context, it is interesting to note that the best annotated animal ortholog, the *Drosophila* protein ZUC (Uniprot Id Q9VKD7), is associated with the mitochondria making an introduction of this gene into the eukaryotes via the endosymbiont conceivable.

### Generation and Fitness Analyses of *pld* Mutants

In order to facilitate the functional characterization of the various PLDs we established a two-step procedure for markerless mutagenesis of *A*. *baumannii*. This procedure is favorable since it avoids polar effects. In the first step we performed insertion duplication mutagenesis of the target *pld* genes using a recombinant pBIISK plasmid, which carries a kanamycin resistance gene (*kanR*) as positive selection marker, a levansucrase gene (*sacB*) for negative selection and 1500 bp of the upstream and downstream region flanking the *pld* gene of interest. With this technique a complete set of phospholipase D single (Δ*pld1*, Δ*pld2*, Δ*pld3)*, double (Δ*pld1+2*, Δ*pld1+3*, Δ*pld2+3*) and triple (Δ*pld1-3*) mutants was generated. All mutants were verified by sequencing using the primer combination ctr_for and ctr_rev (S3 Table).

We determined whether the knockout of the phospholipases had any general impact on the growth of the mutants by comparing the growth on LB and mineral medium (MM) of all *pld mutants* to that of the wild-type. Notably, the WT and the mutants reached a comparable final OD_600_ of 3.3 to 3.5 in LB-medium with a growth rate of 0.9 to 1.0 h^-1^ and a maximal OD_600_ of 1.2 to 1.3 in MM + 20 mM acetate, with a growth rate of 0.75 to 0.80 h^-1^, indicating that knock out of any of the *plds* had no effect on general fitness. Next we raised the question whether PLDs are required for growth on PC. Therefore we tested growth of the Δ*pld1-3* triple mutant on PC as sole carbon source. No difference in growth rate and final cell density was observed compared to the wildtype, leading to the conclusion, that these PLDs are not essential for growth on PC.

### Phospholipases D are not essential for Interbacterial Competition

**Fig 5:**
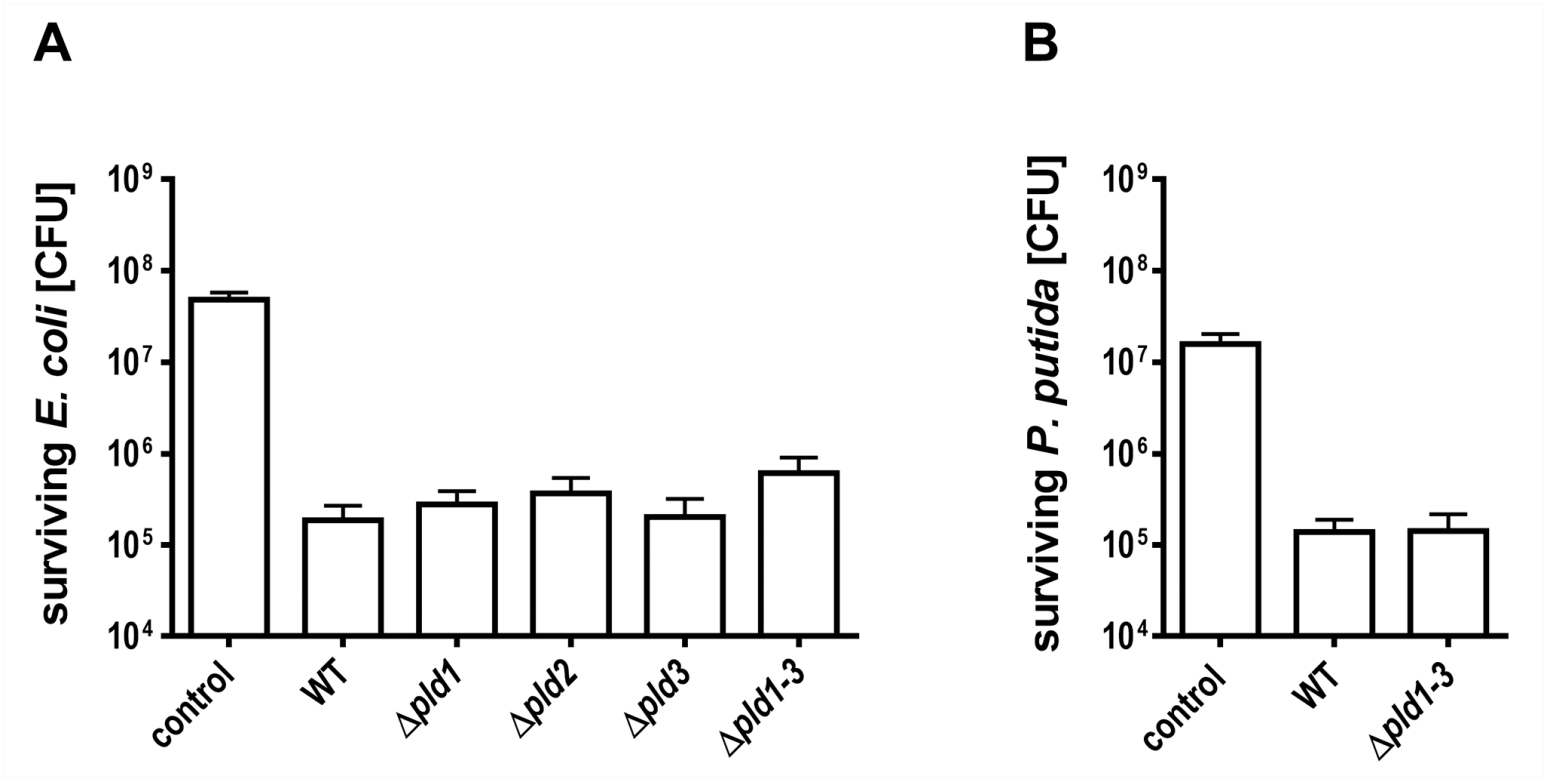
Bacterial competition between *A*. *baumannii* ATCC 19606^T^ and *E*. *coli* or *P*. *putida*. *A*. *baumannii* ATCC 19606^T^ wild-type or *pld* mutant cells and *E*. *coli* DH5α pET28a cells (A) or *P*. *putida* 548.C8 cells (B) were mixed at a ratio of 10:1, spotted onto an LB-agar plates and incubated for 4 hours at 37°C. To quantify the number of surviving *E*. *coli* or *P*. *putida* cells serial dilutions were plated onto kanamycin containing LB-agar and colony forming units were counted after incubation overnight at 37°C or 30°C, respectively. The standard deviation was calculated from three independent experiments. LB medium was used instead of *A*. *baumannii* as negative control.

Recently, it was shown that PLDs of *Pseudomonas aeruginosa* are implicated in bacterial competition [47]. Therefore we addressed the question whether *A*. *baumannii* ATCC 19606^T^ outcompetes other bacteria. We used *E*. *coli* as model strain in a bacterial competition assay as described earlier for competition between *A*. *nosocomialis* and *E*. *coli* [41,42]. *A*. *baumannii* ATCC 19606^T^ wild-type cells were mixed with kanamycin resistant *E*. *coli* in a 10:1 ratio. A mixture of LB-medium and *E*. *coli* in the same ratio was used as negative control. After incubation of the mixture for 4 hours, the colony forming units of *E*. *coli* cells were determined by plating serial dilutions onto kanamycin containing agar plates. Incubation with *A*. *baumannii* ATCC 19606^T^ wild-type revealed 1.9∙10^5^ ± 0.8∙10^5^ colony forming *E*. *coli* cells (Fig 5A). Incubation of *E*. *coli* cells with LB medium as control led to the detection of 4.9∙10^7^ ± 0.9∙10^7^ CFU. This provides clear evidence that *A*. *baumannii* ATCC 19606^T^ outcompetes *E*. *coli*. To verify our results we repeated the competition analyses using a kanamycin resistant *Pseudomonas putida* strain 548.C8 [48]. 1.4x10^5^ ± 0.5∙10^5^ *P*. *putida* CFU were determined when incubated with *A*. *baumannii* wild-type cells, which clearly shows that *A*. *baumannii* also outcompetes *P*. *putida* (Fig 5B).

Apparently, the *pld* single mutants were not affected in outcompeting *E*. *coli* (Fig 5A). The same was observed for the triple mutant which was also not affected in outcompeting *E*. *coli* or *P*. *putida*. 6∙10^5^ ± 2.9∙10^5^ *E*. *coli* and 1.4∙10^5^ ± 0.7∙10^5^ *P*. *putida* cells were counted after incubation with the *pld* triple mutant (Fig 5B). As control living cell counts of *A*. *baumannii* WT and Δ*pld1-3* mutant were analyzed by plating dilutions on LB agar + ampicillin for selection of *A*. *baumannii*. This control supported our finding since no difference in CFU/ml was observed between wildtype and Δ*pld1-3* mutant after competition with either *E*. *coli* or *P*. *putida*. ~ 2.6∙10^8^ CFU/ml of WT or Δ*pld1-3* mutant survived when incubated with *E*. *coli* and ~ 2.1∙10^8^ CFU/ml survived when incubated with *P*. *putida*. Our findings are consistent with the notion, that *A*. *baumannii* PLDs are not essential for antibacterial activity against *E*. *coli* or *P*. *putida* under the conditions tested.

### Identification of Phospholipases D as Virulence Factors *in vivo*

**Fig 6:**
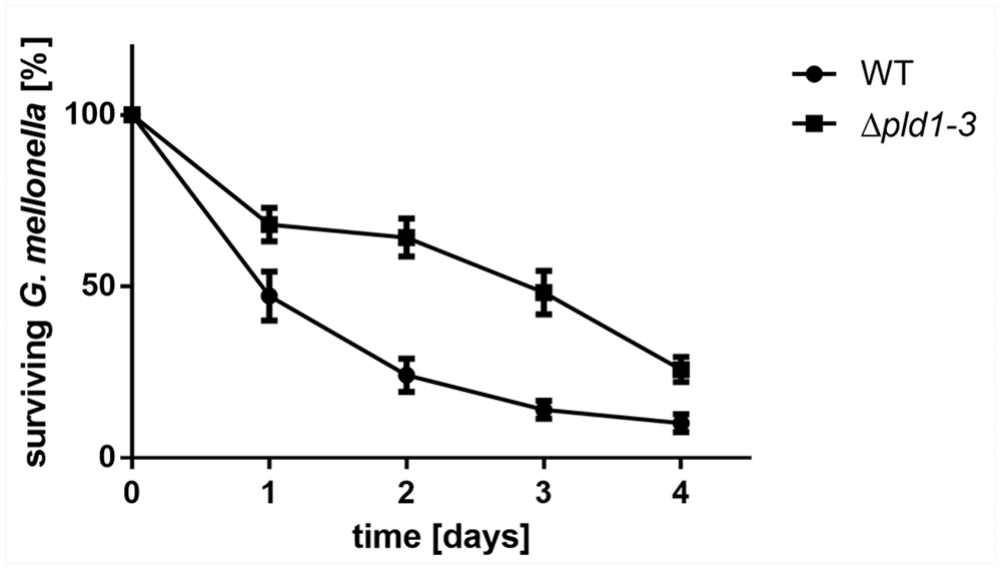
*A*. *baumannii* Δ*pld1-3* mutant is less virulent in *G*. *mellonella* infection. For infection assays 16 caterpillars were injected with approximately 10^6^ bacteria and incubated at 37°C in the dark. Mean difference between the wild-type and the Δ*pld1-3* mutant in four independent experiments was significant after 1, 2, 3 and 4 days (paired t-test; p<0.05 for day 1, p<0.01 for days 2, 3 and 4). As control served an untreated set of caterpillars as well as a set of caterpillars which was treated with 10 μl of sterile 0.9% NaCl. Tests in which more than 2 caterpillars in one of the control groups died after 4 days were not considered.

**Fig 7:**
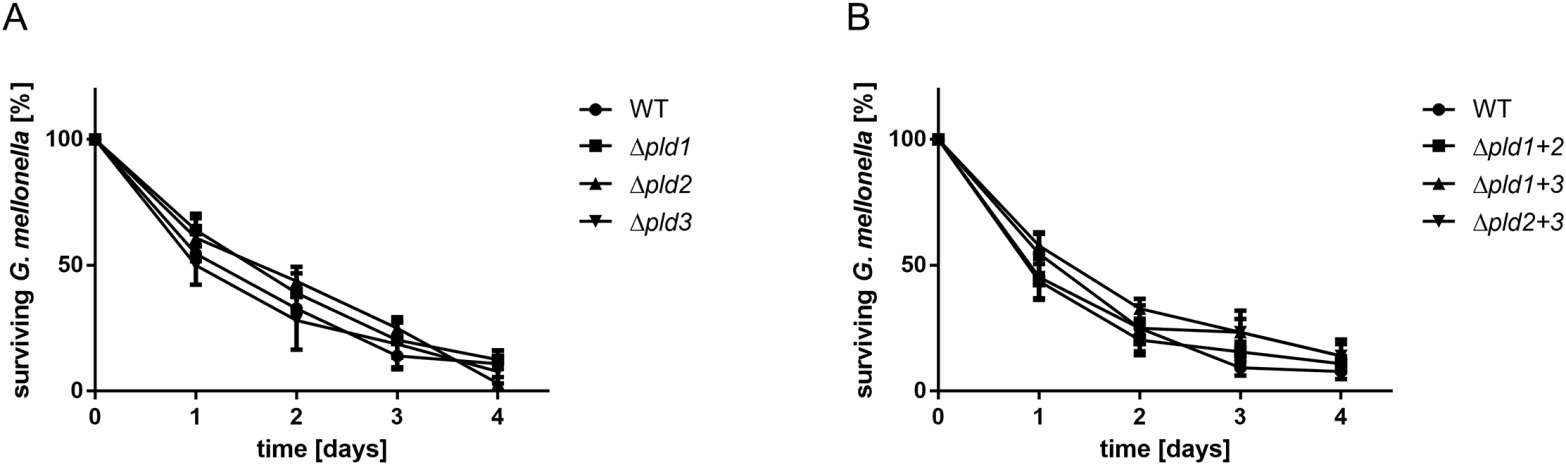
Single or double *pld* mutants display an unaffected virulence towards *G*. *mellonella*. For infection assays 16 caterpillars were injected with approximately 10^6^ bacteria and incubated at 37°C in the dark. Mean difference between wild-type and single (A) or double mutants (B) was not significant. As control served an untreated set of caterpillars as well as a set of caterpillars which was treated with 10 μl of sterile 0.9% NaCl. Tests in which more than 2 caterpillars in one of the control groups died after 4 days were not considered.

The larvae of the greater wax moth (*Galleria mellonella*) have previously been described as a suitable model system to study *Acinetobacter* pathogenesis *in vivo* [49]. In particular, strain ATCC 19606^T^ was shown to kill *G*. *mellonella* in a time and dose dependent manner [13]. To elucidate the role of *A*. *baumannii* PLDs in the infection process, *G*. *mellonella* caterpillars were infected with 10^6^ cells of *A*. *baumannii* ATCC 19606^T^ wild-type or the Δ*pld1-3* mutant by injecting the bacterial cell suspensions (10 μl, OD_600_ = 2) into the hemolymph via the last left proleg. Infected caterpillars were incubated at 37°C in the dark. The *A*. *baumannii* wild-type killed the caterpillars in a time dependent manner (Fig 6). 52.8 ± 7.2%, 75.9 ± 4.8%, 84.4 ± 3.1% and 89.8 ± 2.6% of the caterpillars were dead 1, 2, 3 and 4 days after infection. In contrast infection with the *pld* triple mutant led to a significant reduction in the mortality of the caterpillars compared to the wild-type (paired t-test; p<0.05 for day 1; p<0.01 for days 2 to 4). Only 31.9 ± 4.9%, 35.7 ± 5.6%, 51.56 ± 6.4% and 74.2 ± 3.6% of larvae were dead after 1, 2, 3 and 4 days of infection, respectively (Fig 6). Subsequently, we addressed the question whether the observed virulence deficiency can be linked to the inactivation of individual phospholipases D. We repeated the *G*. *mellonella* infection assay with all single and double *pld* mutants. Interestingly, caterpillars infected with either *pld* single or double mutants died with a similar rate as caterpillars infected with the wild-type (Fig 7). The finding that only the *pld* triple mutant exhibited reduced virulence in the *Galleria* infection implies that the concerted action of all three phospholipases are necessary for full virulence of *A*. *baumannii* ATCC 19606^T^ in a *Galleria* model.

### Full Invasion Efficiency requires the concerted Action of all Three PLDs

**Fig 8:**
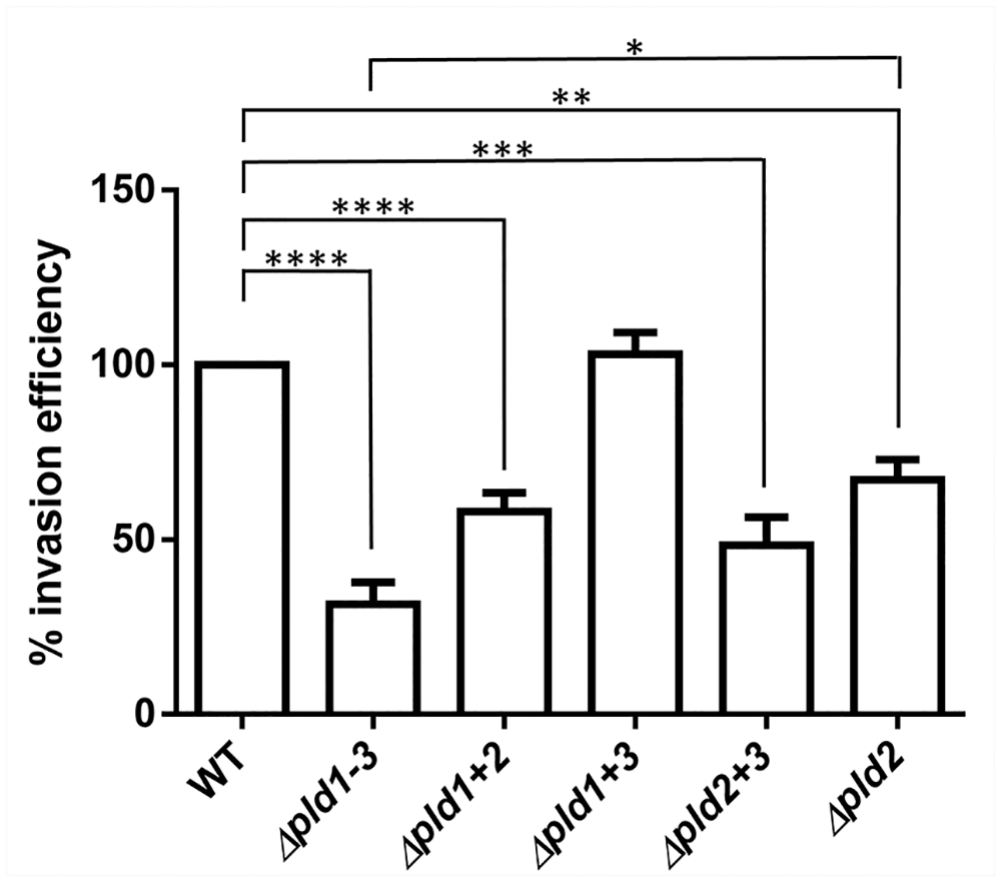
PLDs affect invasion efficiency of *A*. *baumannii*. A549 cells were infected with *A*. *baumannii* wild-type or mutant cells for 5 hours. Then cells were treated with gentamycin to eliminate all external bacteria, prior to release of internal bacteria by lysis with H_2_O. Internal bacteria were calculated and the cell count of the wild-type was set to 100%.100% corresponds to 112.5 ± 2.5 CFU/ml Asterisks indicate statistical significance by paired t-test; *p<0.05; **p<0.01; ***p<0.001; ****p<0.0001.

To analyze the role of the PLDs in human host cell invasion, we tested the *pld* mutants for invasion efficiencies in a gentamycin protection assay, as described earlier [43]. Single *pld1* and *pld3* mutants had no effect on invasion efficiency, whereas a single *pld2* mutant reduced the invasion rate by 32.8 ± 5.7%. A reduction in invasion by a *pld2* mutant of *A*. *baumannii* had actually been described before [12]. A double knock-out of *pld1+2* or *pld2+3* reduced the rate even more and the most reduced rate was observed in the Δ*pld1-3* triple mutant (Fig 8). This finding suggests, that the concerted action of all three PLDs contributes to host cell invasion.

Note that the observed reduction of internal bacteria of the Δ*pld1-3* mutant could also be caused by a deficiency of this mutant to adhere to the A549 cells. To test this possibility we additionally performed adhesion assays. This assay revealed that the Δ*pld1-3* mutant is not impaired in cell association to A549 cells compared to the wild-type (105.5 ± 14.57% compared to the wildtype).

## Discussion

Here, we report on the identification and characterization of three phospholipases D (PLDs) in *A*. *baumannii* ATCC 19606^T^: PLD1, PLD2 and PLD3. None of the three PLDs was required for growth on PC. However, our finding, that palmitate, a PC cleavage product, liberated from PC via phospholipase A (PLA) activities suggests, that a PLA might be implicated in growth on PC. This is supported by the detection of a highly conserved phospholipase A (HMPREF0010_00411) in the genome of *A*. *baumannii*.

The fact that multiple phospholipases D are present throughout the genus *Acinetobacter* (S2 Table) together with our finding that all PLDs play an important role in virulence and host cell invasion suggests, that PLDs are major virulence factors of *A*. *baumannii*. Two of these genes, *pld1* and *pld2* share a conserved domain architecture (two PLDc_2 domains) and are considerably close paralogs that emerged within contemporary *Moraxellaceae* and presumably in the last common ancestor of *Acinetobacter*. The same domain architecture is seen across a broad spectrum of bacterial orthologs suggesting that it represents the archetype of bacterial PLDs [50]. The third PLD, PLD3 differs from this scheme. Although similar in size, the encoded PLD3 protein harbors only a single PLDc_2 domain located in the C-terminal half of the protein with an active site having the functionally relevant aspartic acid residue (D) replaced by a tyrosine (Y). The functional consequence of this D to Y replacement, which is specific for the PLD3s of the genus *Acinetobacter* (S4 File), remains unclear and is subject to future studies. Notably, a second active site motif is located in the N-terminal half of the protein, although the corresponding PLDc_2 domain appears absent. Our finding is of particular interest since a general feature of all known members of the PLD superfamily is, that two HKD motifs are required for the catalytic activity and that all conserved residues of both HKD motifs are essential for PLD activity [24,45,51]. Even a single mutation of one aspartate residue leads to an almost complete loss of catalytic activity in mammalian PLD1 [52] or even to an insoluble protein such as in the case of *Yersinia pestis* murine toxin, which represents another member of the PLD superfamily [53]. It will now be interesting to see whether a non-conserved lysine residue detected next to the tyrosine residue, an aspartic acid downstream of the HKD-motif, or the tyrosine itself can take over the function of the otherwise highly conserved aspartic acid residue in the active site. In this instance, it can be speculated that the modified motif might affect functionality thereby modifying the activity of this phospholipase. Alternatively, the hydrolysis could be accomplished by the second active site motif displaying the canonical D. As, however, two fully functional copies of the active site motif are required for formation of the catalytic core, this would require (homo-) dimerization of PLD3. It was shown on the example of the PLD3 ortholog ZUC from *Drosophila melanogaster*, that this protein indeed is capable of exerting a double functionality. ZUC acts both as a phospholipase hydrolyzing cardiolipin and also as a nuclease, which is involved in the formation of small functional RNAs implicated in the PIWI pathway (piRNAs) [54,55]. From this aspect, it wouldn’t be too surprising if also PLD3 displays an activity beyond the cleavage of phosphatidylcholine. It will be interesting to see whether this is indeed the case, and to what extent PLD3 might even resemble the functional role as a nuclease described for its metazoan orthologs ZUC.

Each of the three PLDs was found to contribute to virulence in the eukaryotic *G*. *mellonella* infection model and invasion of human lung epithelial cells. These findings imply that the PLDs of *A*. *baumannii* are important effectors mediating virulence and invasion in eukaryotic host cells. *pld2* corresponds to the recently identified *pld* gene in the clinical isolate 98-37-09. Disruption of this *pld* gene in this isolate via transposon mutagenesis resulted in reduced growth of the isolate in human serum and a decrease in invasion of BEAS-2B bronchial epithelial cells and HeLa cervical cancer cells [12]. Furthermore a reduction in bacterial burden of blood, heart and liver was found. In agreement with the findings by Jacobs *et al*. [12] our studies revealed that a single *pld2* mutation led to a statistically significant reduction in invasion efficiency using A549 lung epithelial cells. However, only upon inactivation of all three *pld* genes invasion efficiency reached a minimum, indicating that the concerted action of all three PLDs was necessary for full invasion and virulence. From an evolutionary perspective, one observation in this context is particularly interesting. While the sole knockout of *pld2* markedly decreases the capability for cell invasion, no effect is seen when knocking out its close paralog *pld1*. Given the considerably recent evolutionary separation of *pld1* and *pld2*, most likely in the common ancestor of *Acinetobacter*, this indicates a neo-functionalization of either protein. One indication for such a functional change comes from the domain architecture of both proteins (Fig 2). PLD1 lacks a membrane anchor that is present in PLD2. As a consequence, both proteins are likely to differ in their cellular localization.

Future studies will show, whether the differences observed between the different *pld* mutants are caused by functional changes such as different substrate specificities and/or different modes of regulation. This would be in line with previous observations, which showed that PLDs not only mediate cleavage of phosphodiester bonds in phospholipids but some also hydrolyze polynucleotide backbones or exhibit phosphatase activity [24,55]. The latter might also contribute to phosphate scavenging of pathogenic bacteria. In this context, a PLD mediated release of phosphate from PC could be beneficial for invasion of the human host via wound infections. It is very interesting to note, that strain ATCC 19606^T^ has been reported to have a reduced ability to metabolize phosphate compounds [56] and it is tempting to speculate, that PLDs might also play a role in phosphate acquisition. Whether this is indeed the case and whether there is even a link between PLDs and the phosphate (*pho*) regulon found in *A*. *baumannii* will be addressed in future studies. Furthermore it will be interesting to see whether the three PLDs possess an active role in the internalization process. The latter has been shown for PLDs of *Neisseria gonorrhoeae* and *P*. *aeruginosa*, which directly interact with host kinases recruited by the pathogens to enter epithelial cells [47,57]

Moreover, PLD activities generate bioactive molecules, such as phosphatidic acid from the hydrolyzation of phospholipids, potentially affecting eukaryotic signal cascades. The latter has already been shown in gonococci, *P*. *aeruginosa* and *Streptomyces chromofuscus* [47,58,59] where the PLD mediated cleavage of lysophosphatidylcholine (LPC) triggers signaling cascades via G-protein-coupled receptors in host cells. The contribution of these distinct features to the three PLDs of *A*. *baumannii* will be deciphered in future studies.

## Supporting Information

S1 Fig Pfam domain architecture of the PLD3 protein family. The tree topology represents the one shown at S3 File. The overview reveals that most of the bacterial PLD3 members and all of the eukaryotic PLD3 orthologs share the presence of only a single PLDc_2 domain.
(PDF)

S1 Table TOL taxa set. Comprising 1606 taxa. (XLS)

S2 Table ACI taxa set. The set of 68 analyzed Acinetobacter taxa and the respective presence/absence of PLD orthologs. Percent identity values are given in parentheses. * denotes that the protein was not present in the protein collection of the data source.
(XLS)

S3 Table Primer for markerless mutagenesis. (DOCX)

S4 Table Phyletic pattern of PLD orthologs across the set of 1,608 analyzed taxa. (DOCX)

S5 Table Taxa set for maximum likelihood tree reconstruction of PLD1 and PLD2 orthologs. (DOCX)

S1 File Orthologous and co-orthologous sequences with given protein ID, taxon name, assignment and amino acid sequence. (XLSX)

S2 File Tree topology for 1180 taxa representing the tree for all three PLD orthologs. (NEXUS)

S3 File Tree topology for 246 taxa representing the tree of PLD3 orthologs. (NEXUS)

S4 File Alignment of HKD motif. Alignment of HKD motif for a selection of 238 bacterial and archaeaen taxa including 68 *Acinetobacter* strains with the unusual HKD-like motif with given taxon name and amino acid sequence.
(XLSX)
