# Supplementary figures and images for: Acinetobacter baumannii Virulence Is Mediated by the Concerted Action of Three Phospholipases D
Source: PLoS One. 2015 Sep 17;10(9):e0138360. doi: 10.1371/journal.pone.0138360 (PMC4574555; doi:10.1371/journal.pone.0138360)

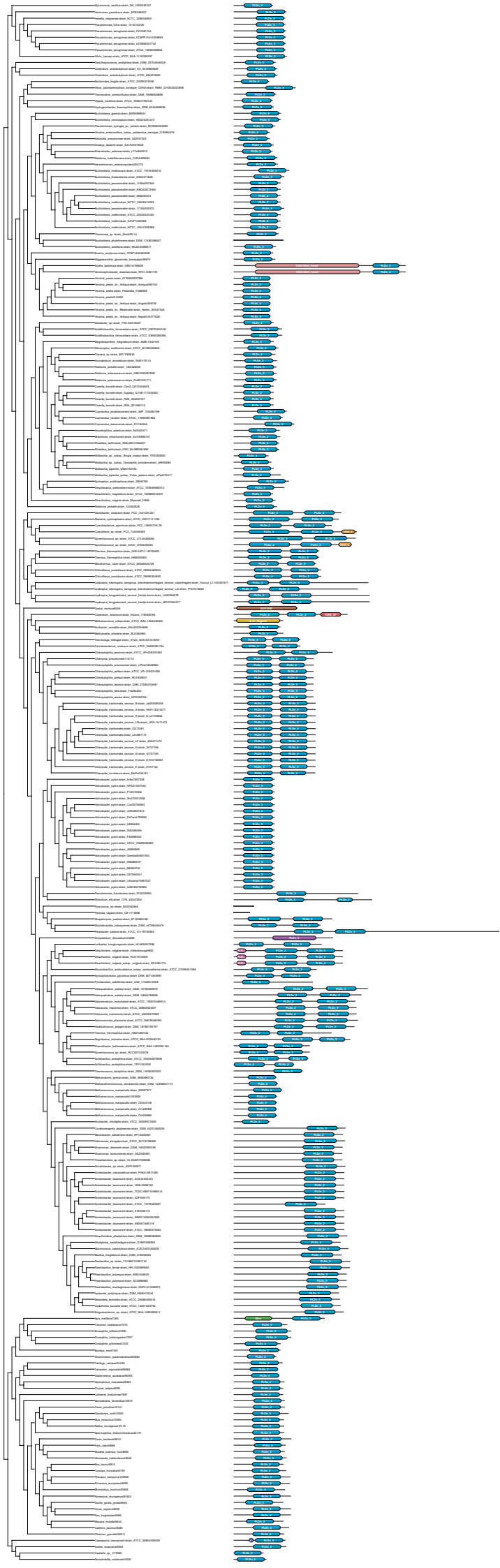

Supplement: S1 Fig — The tree topology represents the one shown at S3 File. The overview reveals that most of the bacterial PLD3 members and all of the eukaryotic PLD3 orthologs share the presence of only a single PLDc_2 domain. (PDF) [file pone.0138360.s001.pdf]
