# Supplementary material for: Acinetobacter baumannii Virulence Is Mediated by the Concerted Action of Three Phospholipases D
Source: PLoS One. 2015 Sep 17;10(9):e0138360. doi: 10.1371/journal.pone.0138360 (PMC4574555; doi:10.1371/journal.pone.0138360)
Supplement: S3 Table — (DOCX) [file pone.0138360.s004.docx]

**S3 Table. Primer for markerless mutagenesis.**

| **Primer** | **Sequence 5`-3`** | **Application** |
| --- | --- | --- |
| *SacB*/Kan_for | GGAAAGCCACGTTGTGTCTC | Amplification of the *sacB/kanR* cassette |
| *SacB*/Kan_rev | GCTCTGCCAGTGTTACAACC | Amplification of the *sacB/kanR* cassette |
| pld2_ for_KpnI | ggggtaccCCTGCACGAATTATACTCTAGC | Cloning of *pld2* (HMPREF0010_03706) in pBIISK |
| pld2_ rev_PstI | aactgcagAACCAATGCATTGGACTTACACTTCCTCCTATTTC | Cloning of *pld2* (HMPREF0010_03706) in pBIISK |
| pld2_ctr_for | AAACGGCGATCTGGATTTAAACGATATTGGTGAAC | Verification of *pld2-*mutant |
| pld2_ctr_rev | CTGCTATCGAAGCTCAGCGTCCGGTACAGGTATG | Verification of *pld2-*mutant |
| Pld1_up_for_PstI | GCCGCTGCAGTTCTCTGACTTCATCATGTGAAATATC | Cloning of the upstream region of *pld1* in pBIISK_*sacB/kanR* |
| pld1_up_rev_ BamHI | GCGCGGATCCTGCCTCATCATTCAACCAATTATTTGTC | Cloning of the upstream region of *pld1* in pBIISK_*sacB/kanR* |
| pld1_do_for_ BamHI | CGGCGGATCCCTCTTATTTGCCAATTGAGTGGATGATG | Cloning of the downstream region of *pld1* in pBIISK_*sacB/kanR* |
| pld1_do_rev_ NotI | TAACGCGGCCGCTAGTATTAGGAACAGGTATTGCGATTGG | Cloning of the downstream region of *pld1* in pBIISK_*sacB/kanR* |
| pld1_ctr_for | TTGAGCTTATCTTTAATTAGCTTAGTATG | Verification of *pld1-*mutant |
| pld1_ctr_rev | AGCGGCTCAAATGAAAGCCCTTAAATAAC | Verification of *pld1-*mutant |
| pld3_up_for_PstI | cgtactgcagGCAGCCAAGTTGCTTTACGTGTTGAG | Cloning of the upstream region of *pld3* in pBIISK_*sacB/kanR* |
| pld3_up_rev_ BamHI | gctaggatccACCGCCATATAACGACGTCCTGACC | Cloning of the upstream region of *pld3* in pBIISK_*sacB/kanR* |
| pld3_do_for_ BamHI | gcgaggatccGCCGATGAGTCTAAAGTGAAGTATTGG | Cloning of the downstream region of *pld3* in pBIISK_*sacB/kanR* |
| pld3_do_rev_ NotI | gtcggcggccgcATTTATGTCGCCATTACAGCCCTAAC | Cloning of the downstream region of *pld3* in pBIISK_*sacB/kanR* |
| pld3_ctr_for | AACTGGCGAGCGCTGGCGTAACTGTTGG | Verification of *pld3-*mutant |
| pld3_ctr_rev | GTATGATTGCTTGGTCTGCTGCTCATAC | Verification of *pld3-*mutant |
