# Supplementary material for: Acinetobacter baumannii Virulence Is Mediated by the Concerted Action of Three Phospholipases D
Source: PLoS One. 2015 Sep 17;10(9):e0138360. doi: 10.1371/journal.pone.0138360 (PMC4574555; doi:10.1371/journal.pone.0138360)
Supplement: S4 Table — (DOCX) [file pone.0138360.s005.docx]

**S4 Table. Phyletic pattern of PLD orthologs across the set of 1,608 analyzed taxa.**

|  |  | **PLD1** | **PLD2** | **PLD3** |
| --- | --- | --- | --- | --- |
| **Bacteria (1281)** | **Proteobacteria (621)** | 245 | 278 | 118 |
|  | **Chlamydia (22)** | 2 | 19 | 20 |
|  | **Firmicutes (264)** | 181 | 96 | 17 |
|  | **High GC Groups (135)** | 11 | 15 | 3 |
|  | **Cyanobacteria (41)** | 2 | 0 | 7 |
|  | **CFB Group (49)** | 18 | 13 | 3 |
|  | **Thermotogales (9)** | 1 | 0 | 2 |
|  | **GNS (13)** | 0 | 0 | 2 |
|  | **Spirochetes (29)** | 6 | 1 | 4 |
|  | **Fusobacteria (5)** | 2 | 2 | 3 |
|  | **Verrucomicrobia (4)** | 3 | 1 | 1 |
|  | **Aquificales (7)** | 0 | 0 | 1 |
|  | **Mycoplasmas (38)** | 10 | 7 | 0 |
|  | **green sulfur bacteria (11)** | 0 | 0 | 0 |
|  | **Planctomycetes (2)** | 2 | 0 | 0 |
|  | **Other (31)** | 5 | 1 | 6 |
| **Archaea (123)** |  | 3 | 3 | 18 |
| **Eukaryota (204)** | **Unikonta** | 1 | 0 | 41 |
|  | **Bikonta** | 6 | 0 | 0 |
|  |  | 498 | 436 | 246 |
