# Supplementary material for: Acinetobacter baumannii Virulence Is Mediated by the Concerted Action of Three Phospholipases D
Source: PLoS One. 2015 Sep 17;10(9):e0138360. doi: 10.1371/journal.pone.0138360 (PMC4574555; doi:10.1371/journal.pone.0138360)
Supplement: S5 Table — (DOCX) [file pone.0138360.s006.docx]

**S5 Table. Taxa set for maximum likelihood tree reconstruction of PLD1 and PLD2 orthologs.**

| **Taxon lable** | **Uniprot Entry** |
| --- | --- |
| PLD1/2 Vibrio fischeri ATCC 700601 / ES114 | Q5E4E5_VIBF1 |
| PLD1/2 Psychrobacter cryohalolentis K5 | Q1Q9Z4_PSYCK |
| PLD1/2 Psychrobacter arcticus DSM 17307 / 273-4 | Q4FRF9_PSYA2 |
| PLD1/2b Psychrobacter sp. PRwf-1 | A5WDS6_PSYWF |
| PLD1/2a Psychrobacter sp. PRwf-1 | A5WEH2_PSYWF |
| PLD1/2a Pseudomonas aeruginosa PA7 | A6VEK6_PSEA7 |
| PLD1/2 Pseudomonas putida F1 / ATCC 700007 | A5WAZ4_PSEP1 |
| PLD1/2b Pseudomonas aeruginosa PA7 | A6VEB6_PSEA7 |
| PLD2 Acinetobacter baylyi ATCC 33305 / ADP1 | Q6F8A6_ACIAD |
| PLD2 Acinetobacter baumannii TCDC-AB0715 | F0QGW2_ACIBD |
| PLD2 Acinetobacter baumannii ATCC 19606 | D0CG26_ACIBA |
| PLD2 Acinetobacter calcoaceticus PHEA-2 | F0KPJ3_ACICP |
| PLD1 Acinetobacter baumannii ATCC 19606 | D0C777_ACIBA |
| PLD1 Acinetobacter baumannii TCDC-AB0715 | F0QER4_ACIBD |
| PLD1 Acinetobacter calcoaceticus PHEA-2 | F0KP57_ACICP |
| PLD1 Acinetobacter baylyi ATCC 33305 / ADP1 | Q6FB53_ACIAD |
